# Magnetic Particle Imaging (MPI): Experimental Quantification of Vascular Stenosis Using Stationary Stenosis Phantoms

**DOI:** 10.1371/journal.pone.0168902

**Published:** 2017-01-05

**Authors:** Sarah Vaalma, Jürgen Rahmer, Nikolaos Panagiotopoulos, Robert L. Duschka, Jörn Borgert, Jörg Barkhausen, Florian M. Vogt, Julian Haegele

**Affiliations:** 1 Clinic for Radiology and Nuclear Medicine, University Hospital Schleswig Holstein, Lübeck, Germany; 2 Research Laboratories, Philips Technologie GmbH Innovative Technologies, Hamburg, Germany; Brandeis University, UNITED STATES

## Abstract

Magnetic Particle Imaging (MPI) is able to provide high temporal and good spatial resolution, high signal-to-noise ratio and sensitivity. Furthermore, it is a truly quantitative method as its signal strength is proportional to the concentration of its tracer, superparamagnetic iron oxide nanoparticles (SPIOs). Because of that, MPI is proposed to be a promising future method for cardiovascular imaging. Here, an interesting application may be the quantification of vascular pathologies like stenosis by utilizing the proportionality of the SPIO concentration and the MPI signal strength. In this study, the feasibility of MPI based stenosis quantification is evaluated based on this application scenario. Nine different stenosis phantoms with a normal diameter of 10 mm each and different stenoses of 1–9 mm and ten reference phantoms with a straight diameter of 1–10 mm were filled with a 1% Resovist dilution and measured in a preclinical MPI-demonstrator. The MPI signal intensities of the reference phantoms were compared to each other and the change of signal intensity within each stenosis phantom was used to calculate the degree of stenosis. These values were then compared to the known diameters of each phantom. As a second measurement, the 5 mm stenosis phantom was used for a serial dilution measurement down to a Resovist dilution of 1:3200 (0.031%), which is lower than a first pass blood concentration of a Resovist bolus in the peripheral arteries of an average adult human of at least about 1:1000. The correlation of the stenosis values based on MPI signal intensity measurements and based on the known diameters showed a very good agreement, proving the high precision of quantitative MPI in this regard.

## Introduction

Medical imaging methods like Magnetic Resonance Tomography (MRI), Computed Tomography (CT), X-ray, and Sonography are currently state of the art in imaging of vascular pathologies. Furthermore, X-ray Digital Subtraction Angiography (DSA) plays a major role in the assessment of cardiac and cerebral vascular pathologies and is the most common technique used to guide cardiac, cerebral, and peripheral vascular interventions.

Because of its very high temporal and spatial resolution and its ability to cover a large field of view (FOV) in a very short time, CT has become one of the most important imaging techniques for cerebral and peripheral vascular diseases, especially in emergencies. Moreover, it is gaining more importance in cardiac imaging as well, especially for the evaluation of coronary heart disease in patients with an intermediate risk profile or for planning of cardiac procedures like interventional valve replacement [1]. While the spatial and temporal resolution of MRI is not as high as in CT, it does not burden the patient with ionizing radiation and even offers the possibility of vascular imaging without contrast agents. In principle, MRI tends to overestimate vascular stenosis, and CT´s otherwise high diagnostic accuracy is impaired in calcified vessels and stents, which can result in an overestimation of stenosis, too [2]. Besides that, accurate quantification of vascular pathologies is possible with both methods; using sophisticated image analysis programs even automated quantification is feasible and routinely used especially for CT. Color-coded duplex sonography (CCS) also offers a reliable assessment of vascular stenosis, occlusions and blood flow, all that without the use of ionizing radiation. Thus, it is one of the standard methods for evaluation of vascular pathologies. However, penetration depth and FOV are limited and the accuracy of the results very much depends on the experience of the examiner. In DSA, quantification of vascular stenoses has to be conducted subjectively or semi-quantitatively by measurements of two-dimensional projection images. Furthermore, DSA also burdens patient and physician with ionizing radiation.

Magnetic Particle Imaging (MPI) is a three-dimensional, tracer based imaging method using magnetic fields to visualize the spatial distribution of superparamagnetic iron oxide nanoparticles (SPIOs) [3]. MPI thereby exhibits a high spatial and a very high temporal resolution, a high signal-to-noise ratio (SNR) and a high sensitivity [4]. MPI still is an experimental method, but commercial scanners are already available for small animal imaging. Currently, one human sized scanner system has already been built and has delivered initial images [5].

In contrast to MRI, CT and DSA, the intensity of the MPI-signal is proportional to the tracer, i.e. the SPIO concentration [4]. Thus, the SPIO concentration can be deduced directly from the MPI signal. As MPI is a true three-dimensional imaging method, the SPIO concentration inside a whole volume is measured in situ, which may be used for vessel visualization and subsequent stenosis quantification.

Because of MPI´s very high temporal resolution, the SPIO concentration can be measured as a function of time for a certain volume of interest, e.g. the heart, in order to quantify organ perfusion and to visualize the supplying vessel system with sufficient spatial resolution. Additionally, given MPI-compatible devices, also real-time MPI-guided cardiovascular interventions might be an attractive option [6, 7].

The proof of principle of in vivo MPI was provided by three-dimensional visualization of the beating heart of a mouse, which demonstrated the potential of cardiovascular imaging with MPI [8]. However, until now, research in MPI has mainly been focused on scanner/hardware-development and design of dedicated SPIO-tracers. MPI-images of vessels or anatomical vessel phantoms have been demonstrated in a few studies [9–11], but missing systematic evaluation of the application of MPI for quantitative cardiovascular imaging.

Thus, the purpose of this study was to assess the potential of MPI in quantifying the extent of stenoses using a vascular phantom model.

## Material and Methods

### Stenosis and reference phantoms

The stenosis and reference phantoms were made of VisiJet® X (UV curable Plastic, 3D Systems, Rock Hill, SC, USA) by 3D Printing technology (3D Printer ProJet 3510 HD Plus, 3D Systems, Rock Hill, SC, USA) at the Institute of Medical Engineering, University of Luebeck, Germany. The printer provides an accuracy of 25–50 μm per 25.4 mm of part dimension [12]. Constructed as circular cylinders they had an outer diameter of 20 mm. The length of these phantoms was 70 mm. Threaded at the outer sides they could be closed with a Polyoxymethylen (POM) cap.

**Fig 1:**
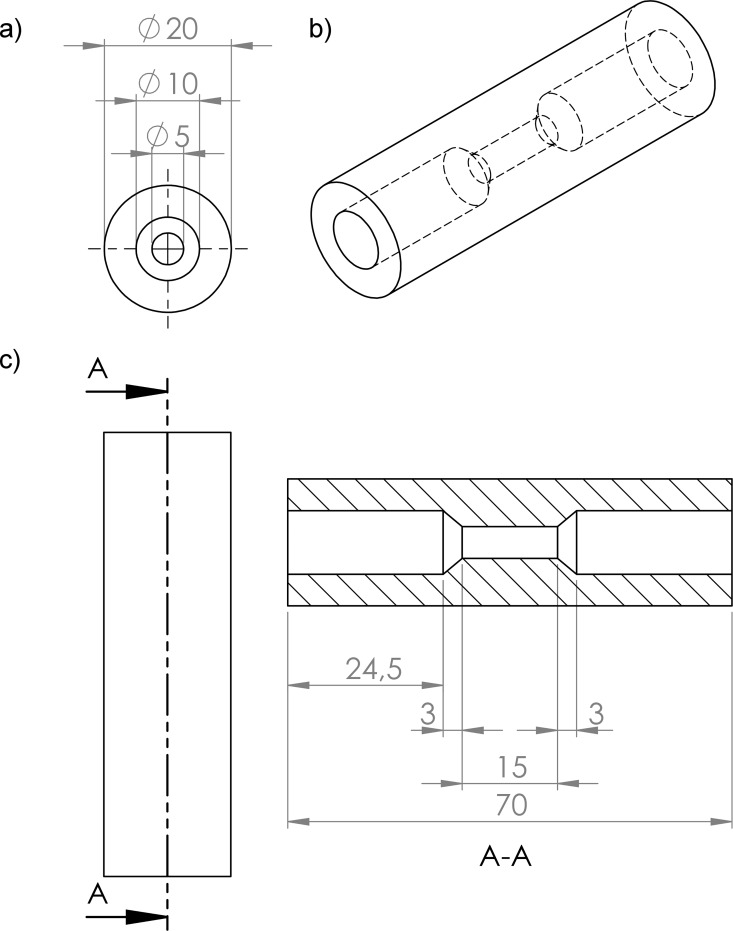
Technical drawing of the 5 mm stenosis-phantom. The stenosis phantom is shown in its cross section (a) and its longitudinal section (c). For better illustration, the stenosis phantom is also shown as an oblique view (b). It can be seen that the stenosis phantom is a cylinder with a length of 70 mm and a diameter of 20 mm. The lumen (not shaded in (c)) consists of three segments: The two outer segments with a length of 24.5 mm and an inner diameter of 10 mm, which was considered the “normal lumen”. The symmetrical central segment with a length of 21 mm with a 3 mm long cone-shaped narrowing at both its endings, which led to the characteristic stenosis, a 15 mm long cylinder with a diameter of 1–9 mm, in this case 5 mm.

Ten different reference phantoms were used; each featured a different continuous inner diameter of 1–10 mm in steps of 1 mm. The nine stenosis phantoms featured a normal lumen with an inner diameter of 10 mm and a characteristic stenosis with a diameter of 1–9 mm according to the used stenosis phantom (Fig 1). This resulted in nine different stenosis diameters: 1 mm diameter amounting to a 99% stenosis of the cross section of the normal diameter, 2 mm (96%), 3 mm (91%), 4 mm (84%), 5 mm (75%), 6 mm (64%), 7 mm (51%), 8 mm (36%) or 9 mm (19%), respectively.

### MPI image acquisition & reconstruction

All stenosis- and reference-phantoms were filled with a 1% dilution of Resovist, amounting to a concentration of 5 mmol (Fe)/l. For image acquisition, each reference- and stenosis-phantom was placed at the center of the bore. Furthermore, to evaluate the ability to quantify stenosis down to very low SPIO-concentrations, a serial dilution of Resovist was measured using the 5 mm (75%) stenosis-phantom. The following dilutions were used: 1:100 (1%, as in the above described measurements), 1:200, 1:400, 1:800, 1:1600, and 1:3200.

MPI image acquisition was performed with an experimental preclinical MPI-demonstrator system (Philips Technologie GmbH, Hamburg, Germany). The drive field was applied on three orthogonal axes with respective amplitudes of 16 mT and frequencies of 25.3 kHz, 24.5 kHz, and 26.0 kHz in z-, x-, and y-direction. This resulted in a volume of 12.8 mm × 25.6 mm × 25.6 mm that was directly encoded by the drive fields. The volume acquisition time was 21.54 ms, amounting to a frame rate of 46.42 Hz. The gradient strength of the selection field was 2.50 T/m in z-direction and 1.25 T/m in x- and y-direction. The phantom was mounted statically at the center of the scanner bore. As the long axis of the phantom (aligned along the bore) extended beyond the volume covered by the drive fields, so-called focus fields were applied to move the imaging volume over the phantom. Focus fields are homogeneous offset fields that are used to increase the total imaging volume by shifting the rather small imaging volume encoded by the drive fields over a larger region in space [13]. For the stenosis measurements, 81 stationary focus field values were applied along the x-axis, shifting the imaging volume over a range from -20.0 to +20.0 mm at steps of 0.5 mm. Each station lasted 2.154 s, so that 100 volumes were acquired per station.

The images were reconstructed over a slightly larger field of view (FOV) than covered by the drive fields, based on a dedicated system function acquired prior to the stenosis measurements. For system function acquisition, a dot-like calibration sample containing 0.8 μl of pure Resovist (Bayer Pharma AG, Berlin, Germany) with a concentration of 500 mmol(Fe)/l was moved with a robot on a grid of 32 × 28 × 24 voxels over a FOV of 35.2 mm × 30.8 mm × 19.2 mm. This resulted in a voxel size of 1.1 mm × 1.1 mm × 0.8 mm at a total of 21504 voxels. At each robot position, the signal was averaged over 20 acquisition cycles to increase SNR. For reconstruction, a row-based iterative algorithm was used to obtain the image [14]. In that process, regularization was applied to adjust the balance between spatial resolution and noise in the image and also a non-negativity criterion was applied to improve image quality based on the knowledge that concentrations cannot be negative. For most data, a regularization factor of λ = 1 delivered a good compromise between SNR and spatial resolution. For image reconstruction of highly diluted samples, where signal quantification and/or visualization of stenosis was unreliable, higher regularization with factors of λ = 10 and λ = 100 were applied to increase the SNR. As mentioned above, a higher λ and thus SNR goes along with a reduced spatial resolution. The quantification measurements and calculations of the reference phantoms and stenosis phantoms were conducted with the standard regularization factor of λ = 1. For the dilution series, the measurements and calculations were also conducted with regularization factors of λ = 10 and λ = 100 to account for the reduced signal to noise ratio (SNR) of the higher Resovist dilutions.

### MPI-image analysis

After image reconstruction, quantitative analysis of the respective image data sets of each phantom was conducted using the program MIPAV (Version 7.0.1, Medical Image Processing, Analysis and Visualization, National Institute of Health, Center for Information Technology, Bethesda, Maryland, USA) [15]. For that, no additional image data had to be acquired.

For quantitative analysis, a two-dimensional region of interest (ROI) was drawn directly around the cross section of each of the 19 phantoms in axial orientation. This ROI was placed in the center of the reconstructed FOV. The average MPI signal intensity was determined over the ROI area. As described above, the FOV was shifted along the long axis of each phantom using focus fields, thus the ROI was moved over the phantom as well. As the focus fields advanced the FOV position by 0.5 mm each 2 s, a time versus average intensity curve was obtained for each phantom. Corresponding to the volume imaging rate, 46 intensity values were acquired per second.

#### Reference phantoms

First, the average intensity values of each of the 10 reference phantoms were calculated over the ROI areas. To evaluate the proposed proportionality of the signal intensity to the SPIOs concentration, the average intensity values (*I*_*av*_) were used to calculate the percentage rate of the relative signal intensity (*I*_*rel*_) of the reference phantoms with a diameter of 1–9 mm in relation to the average intensity value of the 10 mm reference phantom:

$$I_{rel}=\frac{I_{av}\;(reference-phantom\;d=x\;mm)}{I_{av}\;(reference-phantom\;d=10\;mm)}\times 100$$ (1)

This relative signal intensity value for each phantom was compared to the percentage rate of the relative cross sectional area (*A*_*rel*_) of the corresponding reference phantom of 1–9 mm in relation to the 10 mm reference phantom calculated based on their known cross sectional areas (*A*):

$$A_{rel}=\frac{A\;(reference-phantom\;d=x\;mm)}{A\;(reference-phantom\;d=10\;mm)}\times 100$$ (2)

#### Stenosis phantoms

The measured signal intensity values of the stenosis phantoms were plotted as a curve. On the basis of this curve the different sections of the stenosis phantom were identified and for each stenosis phantom the average intensity values of its normal lumen (*I*_*avn*_, representing a diameter of 10 mm) were compared with those of its stenotic section (*I*_*avs*_). The residual signal intensity of the stenosis was indicated as percentage rate, i.e. the relative signal intensity of the stenosis (*I*_*rel*_):

$$I_{rel}=\frac{I_{avs}}{I_{avn}}\times 100$$ (3)

This was conducted for every stenosis phantom. The degree of stenosis was then calculated by subtraction of the relative signal intensity of each stenosis from 100%.

#### Serial dilution

Each dilution was filled into the 5 mm stenosis phantom and then measured. The calculations of the degree of stenosis for each dilution were conducted as described by Eq (3).

#### Signal to noise ratio

The noise of each image data set was determined on a frame measured with the scanner bore empty. From this frame, the average noise per pixel and the standard deviation was calculated using the MIPAV statistics tool (Version 7.0.1, Medical Image Processing, Analysis and Visualization, National Institute of Health, Center for Information Technology, Bethesda, Maryland, USA) [15]. For calculation of the image SNR, the standard deviation of the average noise per pixel was used to preclude bias due to the non-negativity constraint in image reconstruction. The average MPI signal intensity per pixel was determined for each vessel phantom over a volume of interest (VOI) placed at its center, resulting in an SNR according to:

$$SNR=\frac{Average\;MPI-signal\;intensity\;per\;pixel}{SD\;(average\;noise\;per\;pixel)}$$ (4)

### Statistics

The measurements of the reference and stenosis phantoms were compared to each other and to the reference values using the Mann-Whitney-Test; p-values <0.05 were considered significant. In these measurements, a Resovist dilution of 1% (1:100) and a regularization factor of λ = 1 were used. To validate the reliability of this approach and the agreement between calculated and measuremed values, the Bland-Altman method was applied. Both the mean (i.e. the bias) and the standard deviation of the difference between the calculated reference values and measured values of the stenosis and reference phantoms were calculated. As described by Bland and Altman, the 95% limits of agreement were defined as mean value + 1,96 × SD (upper limit of agreement, ULA) and mean value—1,96 × SD (lower limit of agreement, LLA) [16]. The results were visualized using Bland-Altman plots.

## Results

### Visualization

**Fig 2:**
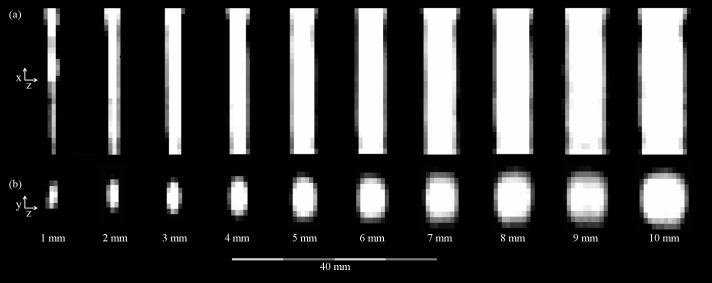
MPI-Images of the reference phantoms. Sagittal (a) and axial (b) slices extracted from reconstructed 3D image data of the reference phantoms. Note the anisotropy of the axial images due to the different gradient field strengths, leading to an oval appearance of the phantoms which is the more pronounced, the smaller the lumen is.

**Fig 3:**
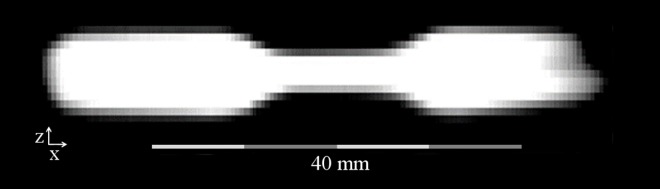
MPI image of the 5 mm stenosis phantom. Image of the 5 mm stenosis phantom reconstructed as covered by the focus fields in z-/x-plane. One end was not fully covered as it extended beyond the encoded volume. It is thus blurry and would not be used in a quantitative evaluation.

**Fig 4:**
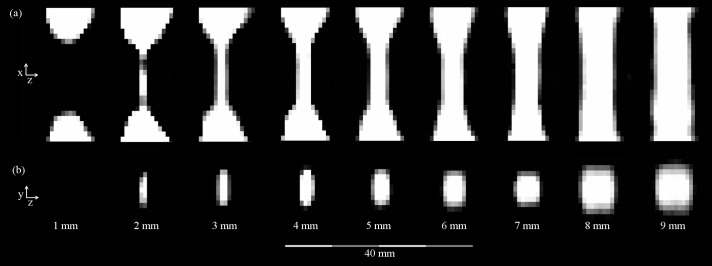
MPI-Images of the stenosis phantoms. Sagittal (a) and axial (b) slices extracted from reconstructed 3D image data of the stenosis phantoms. The lumen of the 1 mm stenosis is not discernible. Note the anisotropy of the axial images due to the different gradient field strengths, leading to an oval appearance of the phantoms which is the more pronounced, the smaller the lumen is.

MPI was able to visualize all straight reference phantoms (1–9 mm Fig 2) and the stenoses of 2–9 mm of the stenosis phantoms (Figs 3 and 4). A spatial resolution of 1.5 mm × 3.0 mm × 3.0 mm was achieved (in z-, x- and y-direction). In opposition to the 1 mm straight reference phantom, the highest grade stenosis of the stenosis phantoms (1 mm stenosis lumen) could not be visually discerned in the MPI images (Fig 4), even with higher regularization factors of λ = 10 and λ = 100, respectively.

There was a distinct anisotropy of the images in y-direction, which resulted in an oval appearance of the phantoms in axial orientation. This was the more pronounced, the smaller the diameter was (Figs 2B and 4B). The anisotropy resulted from the fact that the spatial resolution depends on the gradient strength [17], which is larger in z-direction.

### Quantification

**Fig 5:**
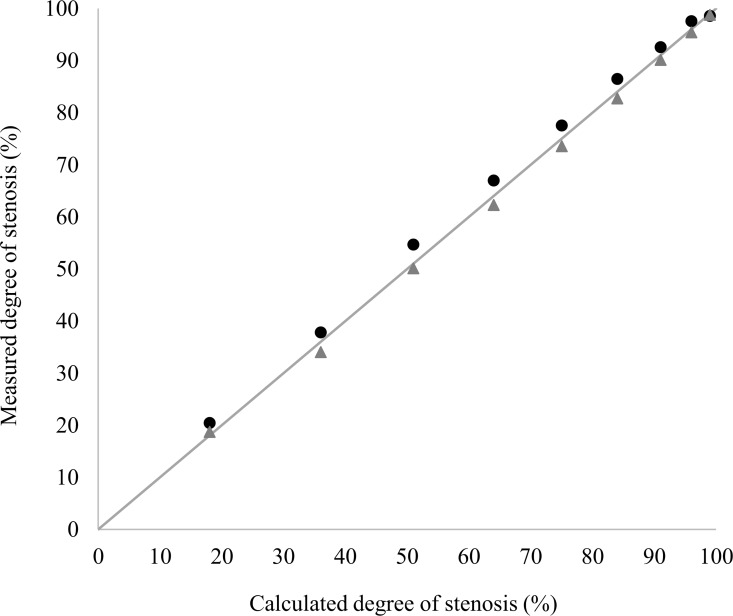
Scatterplot comparing the calculated and measured degree of stenosis. The bisectrix indicates the ideal agreement of calculated (x-axis) and measured (y-axis) values. The values of the stenosis phantoms are indicated as black dots and of the reference phantoms as grey triangles. The good agreement of the values can clearly be delineated. Please also see Table 1, Fig 6 and Fig 7.

Table 1 and Fig 5 show the comparison of the calculated reference values and the measured values of the reference phantoms and stenosis phantoms, respectively.

**Table 1:** Comparison of the degree of stenosis.

Diameter (mm)	Degree of stenosis of:		
	known diameter (%)	reference phantoms (%)	stenosis phantoms (%)
**9**	19	18.72	20.47
**8**	36	34.05	37.84
**7**	51	50.15	54.69
**6**	64	62.31	66.99
**5**	75	73.58	77.59
**4**	84	82.75	86.5
**3**	91	90.16	92.61
**2**	96	95.41	97.58
**1**	99	98.78	98.59

Comparison of the stenosis values calculated based on the known diameters of each stenosis phantom (2^nd^ column), calculated by division of the average intensity of each reference phantom by the MPI signal intensity of the 10 mm reference phantom (3^rd^ column) and calculated by the ratio of average intensities between stenotic and non-stenotic section of each stenosis phantom (4^th^ column). Resovist dilution was 1:100. mm = millimeter, % = percent.

**Fig 6:**
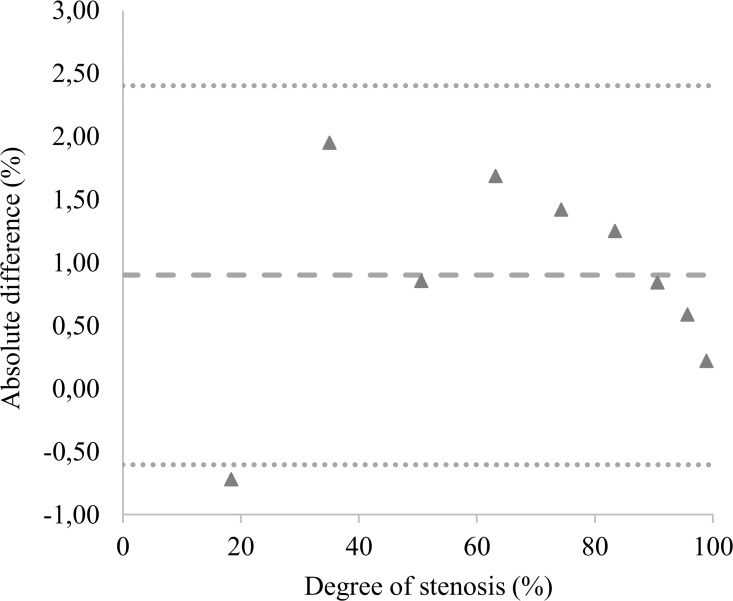
Bland-Altman plot of the difference of the calculated and measured lumen loss of the reference phantom. It can be depicted that the mean absolute difference is 0.9%. Accordingly, the plot shows that there is a slight tendency to underestimate the degree of lumen loss. This effect diminishes with higher degree of lumen loss because of the smaller diameters of the residual lumen.

There was no statistically significant difference between the calculated values and the measured values of the reference phantoms (p = 0.4; Table 1, columns 2, 3; detailed data in S1 Table). The mean difference, i.e. the average absolute bias between the calculated reference values and measured reference phantoms was 0.9% with an upper level of agreement of 2.4% and a lower level of agreement of -0.6% (Fig 6). The Bland-Altman plot shows that the agreement got better with smaller lumina (Fig 6). The mean of the noise level of the measurements of the reference phantoms was 1.71E-05 (SD 1.75E-06). The average MPI-signal intensity per voxel of the small reference phantoms (1–5 mm) was considerably lower than that of the larger reference phantoms (6–10 mm) (detailed data in S5 Table). In detail, the average MPI-signal intensity per voxel increased with increasing diameter of the reference phantoms, but stayed constant for the diameters from 6 to 10 mm. As the noise level was constant, the SNR showed the same behavior as the average MPI-signal intensity per voxel.

**Fig 7:**
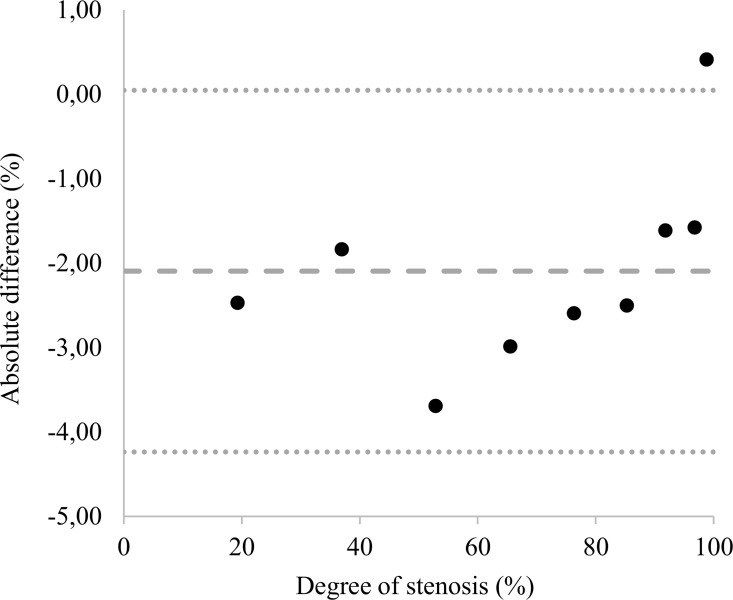
Bland-Altman plot of the difference of the calculated and the measured stenosis of the stenosis phantoms. The mean absolute difference is -2.09%. This indicates that there is a slight overestimation of the degree of stenosis in comparison to the calculated values. This overestimation decreases with increasing degree of stenosis because of the smaller diameters of the residual lumen.

There was also no statistically significant difference between the calculated values and the measured values of the stenosis phantoms (p = 0.4; Table 1, columns 3, 4; detailed data in S2 and S3 Tables). Although the 1 mm stenosis was not discernible in the image due to the large contrast difference, its signal was still detectable in the ROI-based signal intensity evaluation. The mean difference, i.e. the average absolute bias of the calculated reference values and measured stenosis phantoms was -2.9% with an upper level of agreement of 0.05% and a lower level of agreement of -4.24% (Fig 7). The Bland-Altman plot shows that the agreement got better with higher grade stenosis (Fig 7).

The mean of noise level of the measurements of the stenosis phantoms was nearly the same than that of the reference phantom measurements (mean 1.69E-05, SD 2.83E-06 for the stenosis phantoms vs. 1.71E-05, SD 1.75E-06 for the reference phantoms). The mean of the average MPI-signal intensity of the normal lumen (10 mm) of each stenosis phantom was 9.41E-03 (SD 3.91E-04), this resulted in a mean SNR of 570.52 (SD 76.36) (S3 Table).

### Serial dilution

**Fig 8:**
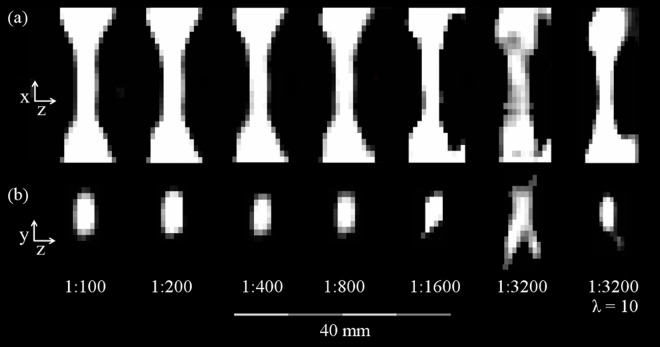
MPI-Images of the dilution series. Sagittal (a) and axial (b) slices extracted from reconstructed 3D image data of the 5 mm stenosis-phantom filled with the different Resovist dilutions from 1:100–1:3200 in sagittal (a) and axial (b) reformations with a regularization factor of λ = 1. Additionally, the image reconstruction of the Resovist dilution of 1:3200 with λ = 10 is shown. Note the distinct distortion of the image of the 1:3200 dilution reconstructed with λ = 1, which are smoothed by the higher regularization of λ = 10 due to the reduced noise (detailed data in S4 Table).

The stenosis of the 5 mm stenosis phantom could be visually discerned with all dilutions, but with the highly diluted Resovist the signal intensity was very low. When using the standard regularization of λ = 1, this led to a very low SNR and image-artifacts in terms of distortions of the 1:1600 and very pronounced of the 1:3200 dilution images (Fig 8). Stenosis quantification using the Resovist dilutions of 1:200–1:1600 showed good agreement with the Resovist dilution of 1:100 and the reference values; it also showed an overestimation of the degree of stenosis (Table 2, detailed data in S4 Table).

**Table 2:** Serial dilution measurements.

Resovist dilution	I_MPI_ of the stenosis	I_MPI_ of the normal lumen d = 10 mm	Relative I_MPI_ of the stenosis^a^ (%)	Degree of stenosis (%)	Signal to noise ratio (SNR)
**1:100**	0.196	0.907	21.621	78.379	454.96
**1:200**	0.099	0.472	20.938	79.062	268.28
**1:400**	0.05	0.228	21.763	78.237	120.72
**1:800**	0.026	0.120	21.639	78.361	76.44
**1:1600**	0.014	0.059	23.321	76.679	31.94
**1:3200**	0.010	0.031	31.519	68.481	4.06
**1:3200**^**b**^	0.007	0.031	21.921	78.078	56.19
**1:3200**^**c**^	0.005	0.027	17.405	82.595	172.66

Comparison of the calculated residual area and degree of the stenosis based on MPI signal intensity measurements of six different Resovist concentrations using the 5 mm stenosis phantom including the SNR of each measurement. The calculated residual area and degree of the stenosis based on the known diameters of the stenosis phantoms are 75% and 25%, respectively. All calculations based on image reconstructions with a regularization factor of λ = 1, unless otherwise indicated. I_MPI_ = MPI signal intensity (arbitrary units), d = diameter, mm = millimeter, mm^2^ = square millimeters, % = percent, SNR = signal to noise ratio; ^a^the relative MPI signal intensity is described in relation to the signal intensity of the normal lumen (d = 10 mm) of the stenosis phantom; ^b^reconstructed with a regularization factor of λ = 10; ^c^reconstructed with a regularization factor of λ = 100 (detailed data in S4 Table).

For λ = 1, the Resovist dilution 1:3200 showed a distinct underestimation of the stenosis (Table 2, S4 Table). As the noise level for all dilution measurements with a regularization factor of λ = 1 was very stable (mean 1.89E-05, SD 2.03E-06), this can be explained by the signal intensity values of the normal lumen and the stenosis. The intensity values of the normal lumen and the stenosis dropped proportionally to the Resovist concentration by half of their value with every step to a higher dilution (1.893–2.070, mean value 1.959, SD 0.062) but with one exception: The intensity value measured inside the stenosis dropped only by a factor of 1.421 from the Resovist dilution of 1:1600 to 1:3200, leading to an underestimation of the 5 mm stenosis measured with a dilution of 1:3200 as the corresponding intensity value of the normal lumen dropped by a factor of 1.935 (Table 2, S4 Table). The irregular behavior of the lowest concentrated sample was related to a very low image SNR (4.06) and disappeared, when the regularization was increased: With a regularization factor of λ = 10, the SNR was increased to 56.19 and the stenosis values were in good agreement with the other dilutions and reference values (Table 2, row 1:3200^b^, S4 and S6 Tables). A regularization factor of λ = 100 led to a SNR of 172.66 but an overestimation of the stenosis with the dilution of 1:3200 (Table 2, row 1:3200^c^, S4 and S6 Tables).

To correlate these results, all other dilutions were reconstructed with λ = 10 and λ = 100 as well. In comparison to λ = 1, for λ = 10 the mean of the noise level for all dilutions decreased to 6.45E-06 (SD 5.11E-07) whereas the average MPI-signal intensity per voxel stayed constant except for the dilution of 1:3200 (see above), resulting in a distinct increase of the SNR for all dilutions (S6 Table). For λ = 100, the mean noise level dropped to 1.78E-06 (SD 1.55E-07), but there was also a slight decrease of the average MPI-signal intensity per voxel (S6 Table). However, the SNR still increased considerably for λ = 100 (S6 Table). For all dilutions except 1:3200, the results of the signal quantification with the standard regularization factor of λ = 1 and the regularization factor of λ = 10 were nearly identical, albeit the SNR was increased (S4 Table). Corresponding to the results of the dilution of 1:3200, the signal quantification with λ = 100 led to a distinct overestimation of all stenoses (for all dilutions MV 82.61%, SD 0.71%, S4 Table). To conclude, a medium regularization using λ = 10 delivered optimal quantification results for all dilutions of the dilution series.

## Discussion

This study confirms the capability of MPI for direct quantitative imaging over a high range of SPIO-concentrations. More importantly, it provides the proof of principle for one possible application of quantitative MPI, i.e. the quantification of vascular stenosis.

### Signal intensity quantification

The degree of lumen loss calculated using the signal intensity measurements of the straight reference phantoms and the stenosis phantoms showed no statistically significant difference from the known reference values. In detail, the Bland Altman analysis showed a very low, positive absolute bias for the straight reference phantoms of 0.9% lumen loss. The values for the 95% limits of agreement were -0.6% for the lower level of agreement (LLA) and 2.4% for the upper level of agreement (ULA). This means that in 95% of all MPI measurements, the measured degree of lumen loss will be at most 2.4% higher or 0.6% lower than the real degree of lumen loss. The positive bias indicates a slight underestimation of the lumen loss.

For the stenosis phantoms, the mean difference was -2.9% stenosis (95% limits of agreement: LLA–ULA: -4.24%– 0.05%). These values suggest a slight overestimation of the extent of the stenosis. This effect seems to get less pronounced with higher degrees of stenosis. This makes sense, as the diameters of the residual lumen decrease and thus the absolute signal intensities decrease as well.

Overall, both the underestimation of the reference phantom lumen loss and the overestimation of the degree of stenosis are very small. Thus, more data are needed to evaluate whether these deviations are a result of a too small sample size, manufacturing tolerances of the phantoms, or are related to the details of the reconstruction process, e.g. the level of regularization.

Independently of MPI system and tracer used, the measurements have to fulfil certain requirements to be reliable. First, the tracer has to be distributed homogeneously in the measured vessel, as otherwise the calculation of the degree of stenosis will not be possible. Differences in intravascular concentration could result in a false degree of stenosis. In principle, there are two different ways to achieve sufficient intravascular contrast, a bolus injection or a long circulating tracer in steady state. If a bolus of tracer is applied, it has to be long enough to achieve a homogeneous concentration within the measured vessel.

Second, the dilution series and SNR-measurements show that noise or artifacts due to background signals are a potential source of error. In detail, the lowest SNR was measured for the Resovist dilution of 1:3200 with a regularization factor of λ = 1, i.e. 4.06 (S4 and S6 Tables). This lead to a distinct overestimation of the extent of stenosis. All other measurements reconstructed with a regularization factor of λ = 1 showed no connection to the SNR, which ranged from 31.94 (Resovist dilution 1:1600, S4 and S6 Tables) to values around 600 (standard measurements with a Resovist dilution of 1:100). This shows that the accuracy of the signal quantification depends on the tracer concentration in a way that the concentration has to be above a certain limit to achieve a sufficient SNR, in our case between 4.06–31.94. Above this limit it seems that the SNR does not influence the results distinctively. It has to be noted that the measurements of the SNR for the reference phantoms below 6 mm were impaired by the limited spatial resolution, as the MPI-signal per voxel was affected by partial volume effects (S5 Table). However, the noise of all measurements of the reference phantoms was rather stable with an SD of about 10%, as was the noise measurements for the stenosis phantoms and dilution series, too. Non-linear effects of the scanner hardware create background signals that vary with time, e.g. due to temperature variations, and explain this SD of about 10% of the noise floor.

As mentioned above, in system function based MPI the signal to noise ratio can also be altered by the regularization factor λ. The grade of regularization is a tradeoff between signal-to-noise ratio and spatial resolution. To reliably quantify the highest diluted Resovist sample of 1:3200, an increased regularization factor of λ = 10 was needed to improve the SNR, whereas for all other concentrations a factor λ = 1 was sufficient. A factor of λ = 100 led to an overestimation of the stenosis as the resulting spatial resolution was no longer sufficient to correctly depict the stenosis. This shows that increasing the regularization factor needs to be done with care, although it improves quantification of low tracer concentrations. In stenoses of unknown extent, which is the normal scenario in clinical routine, a too large λ value could lead to an overestimation of the stenosis.

Our results show, that with adequate regularization, quantification works at a Resovist dilution of 1:3200, i.e. an iron concentration of 156 μmol(Fe)/L. Based on previous works on blood circulation and iodine based contrast agents the peripheral concentration of Resovist after bolus injection in humans *in vivo* can be estimated [18, 19]. In the case of an average adult with a blood volume of 5 L [20] who receives the normal dose of 1.4 mL 500 mmol(Fe)/L Resovist (i.e. a total of 700 μmol Fe = 10 μmol(Fe)/kg body weight) via the cubital vein the bolus of 1.4 mL Resovist is diluted in 500 mL of blood on its way through the pulmonary circulation, i.e. a dilution of about 1:357 (1,4 mmol(Fe)/L). Depending on the distance from the heart to the peripheral artery, an additional dilution can be assumed to be around a factor of 2–5, resulting in a dilution of around 1:1000–1:1500. The higher the distance, the higher the dilution effect and the longer the bolus is spread along the vessel, which is important for a homogeneous distribution. Based on this estimation, our results show that it should be possible to acquire a data set of the human heart, torso or legs with one single peripheral venous injection of Resovist with the currently achieved sensitivity of MPI-systems and without the need to use alternating regularization factors. Also, the bolus in our example should be long enough for a reliable quantification.

In principle, another alternative for bolus admission is the intraarterial injection using a catheter during cardiovascular interventions. If the injection rate is steady and not too close to the site of measurement, a homogenous and highly concentrated bolus can be achieved. However, Resovist is not approved for intraarterial injections. Furthermore, multiple injections would still be needed during such an intervention, which was not intended when Resovist was first approved clinically and thus not evaluated in a clinical context. In the package insert, a hiatus of at least 14 days between two admissions is recommended.

Resovist is not seen as a long circulating for MPI, i.e. a blood pool tracer [21, 22]. That is because Resovist is cleared quickly from the blood stream by liver and spleen. This is especially true for larger particles and particle clusters, which contribute more to the MPI signal of Resovist than its smaller particles [23]. Data in mice using the very sensitive Magnetic Particle Spectroscopy (MPS) show that the signal of Resovist in blood drops below noise level after 15–30 minutes without even reaching a steady state first [22]. After 5 minutes, a maximum of 40% of the initial signal could be measured.

A very important third factor for reliable quantification is the spatial resolution. It has to be high enough to preclude partial volume artifacts. In this context it is very important to remark that in MPI the voxel size does not determine the spatial resolution. The voxel size is determined by the spacing of the grid positions during system function acquisition. If the voxel size is chosen sufficiently small, the spatial resolution in MPI is determined by the applied selection field gradient, the size of the calibration sample and, ultimately, the SPIO characteristics [17, 24, 25]: The steepness of the slope of the SPIO´s non-linear magnetization curve determines the spatial extent to which the signal response is confined and therefore directly determines the achievable spatial resolution. Current SPIOs like Resovist exhibit non-ideal MPI-characteristics, i.e. a low steepness of the magnetization curve. Therefore, a large width of their point-spread function results, so that MPI-signal is smeared and extends beyond the borders of one voxel [17, 26], diminishing spatial resolution and potentially resulting in above mentioned partial volume artifacts. These can e.g. lead to an underestimation of a short stenosis, as the signal of the normal lumen “leaks” into the signal of the stenosis. In our experiments, the spatial resolution was 1.5 mm × 3 mm × 3 mm (z-, x-, y-direction). Thus, to avoid this potential source of error, we constructed phantoms with sufficiently long stenoses (15 mm, Fig 1).

In the end, independent of which tracer is used and especially when using blood pool tracers, it has to be ensured that the MPI signal of the vessel will not be “contaminated” by other tracer containing structures around, i.e. the region of interest has to include only the vessel. This can be achieved even in an automated process, as demonstrated by existing vessel analysis programs, e.g. for CT. Applying these algorithms for MPI, plotting the signal intensity alongside the course of a vessel would allow a rapid quantitative evaluation of a stenosis. Additionally, atherosclerotic plaques, especially those with an inflammatory reaction, can accumulate SPIOs, which may be a source of error in stenosis quantification. As the accumulation process takes up to days [27–29], it may not be a major concern for initial MPI-examinations, but it has to be considered in recurring examinations. On the other hand, the identification of these vulnerable plaques is a very interesting application for vascular MPI itself, as it may help to identify and treat these high risk plaques before a plaques rupture with a subsequent vessel occlusion and organ infarction may occur.

Borgert et al. describe three additional potential sources of error in quantitative MPI [30]: First, missing of the fundamental frequency in the signal. The fundamental component corresponds to an overall offset in the image. It is close to zero for sparse images, where only a fraction of the imaging volume is filled with tracer material. For non-sparse imaging volumes, additional constraints like non-negativity of concentrations [31] or continuity between adjacent FOVs [32] mitigate the problem. As our object is rather sparse and we apply a non-negativity constraint, we assume that in our measurements, the contributions from this mechanism are very small.

Second, like in our experiments, the concentration of SPIOs must be sufficiently low to preclude magnetic interactions between the particles. And third, tracer degeneration has to be considered for in vivo experiments. Although no obvious signal degradation was observed in in vivo experiments [8], this point has to be considered for absolute in vivo quantification. However, as stenosis quantification will mainly rely on a relative signal reduction with respect to the feeding vessels in front of the stenosis, knowledge of the exact absolute particle performance is not mandatory.

### Visualization

The only structure that could not be visualized using MPI was the lumen of the 1 mm stenosis, although the signal intensity measurement was possible and the lumen of the 1 mm straight reference phantom was visible. As described above for the signal intensity curve of the 1 mm stenosis phantom, the reason is the high difference in tracer concentration between the normal lumen and the stenosis. However, the reason the stenosis is still measurable is that the MPI-signal can be detected and thus the integral of the total signal content can be extracted.

Especially the smaller lumina of the phantoms appeared to be oval shaped in axial orientation. This effect can be explained by the anisotropic spatial resolution of 1.5 mm × 3.0 mm × 3.0 mm (in z-, x- and y-direction). For very small stenosis diameters, the anisotropy resulted in a distortion between z-axis on the one and x-/y-axes on the other hand. As the z- and y-axis form the axial slices of the phantoms (Figs 2B and 3B), the distortion was more apparent as in the z-/x-plane (Figs 2A and 4A). The rather low spatial resolution also resulted in blurring of the object structures and an impaired visualization of borders, which e.g. can be seen as a tapering especially along the x-/y-axes of the phantoms. As the effects only lead to a non-optimal spatial assignment of the signal, the total signal content remains quantitatively correct and can be extracted by integration over the signal in an adequately chosen ROI.

### Limitations

This study provides the proof of principle for accurate MPI-based quantification of vascular stenosis. However, as this work is an experimental proof of principle study, there are limitations that have to be addressed.

First of all, the study is limited to data evaluating simple, geometrical stenosis phantoms with a homogeneous distribution of tracer material and without flow. Thus, further experiments are required to show the feasibility of quantitative imaging in dynamic flow experiments, more realistically shaped stenosis phantoms and ultimately in *in vivo* experiments. To achieve valuable results in these more demanding settings, especially two requirements have to be fulfilled: First, the tracer has to be distributed homogeneously. Second, a high enough, ideally submillimeter spatial resolution is necessary to allow a detailed visualization of the vessel and avoid partial volume artifacts in signal quantification. This is especially important in shorter, high grade stenosis and smaller vessels. Nonetheless, a temporal resolution of 21.5 ms was achieved, which would be high enough to resolve dynamic processes.

In our experiments MPI slightly overestimated vascular stenosis, although this observation needs further evaluation with other SPIOs, MPI-systems, reconstruction approaches, and more stenosis models to see if it is really a general issue for MPI.

As discussed above, Resovist is not the ideal SPIO for a high spatial resolution MPI and is not eligible as a blood pool tracer or for multiple injections, which is needed for interventional MPI. However, we decided to use Resovist as it is still the international standard of reference SPIO for MPI and we aimed for reproducible results. Alternative tracers with a better MPI-performance are under development and promising results have already been demonstrated [33, 34]. Another way to create blood pool tracers is to label red blood cells with SPIOs for a very long in vivo circulation time, as has already been demonstrated [35].

The use of dedicated MPI-tracers allowing for a higher sensitivity and spatial resolution should also mitigate the necessity to adjust the regularization parameter for very low SPIO concentrations, which is another limitation of this study. Because at very low SNR or high regularization, the chosen regularization parameter seems to influence the stenosis quantification, a change of λ during a diagnostic or interventional procedure in a clinical scenario is problematic, as it may lead to misleading results in stenosis quantification.

Another limitation is the limited number of data and thus the limited informative value of the statistics. Thus, the very good results shown in this proof of principle study have to be validated in more studies.

### Outlook: MPI for clinical and preclinical imaging

MPI features three-dimensional imaging, a very high temporal resolution and the possibility of quantitative imaging without nephrotoxic contrast agents and ionizing radiation. Due to the high magnetic moment of the SPIO-based tracer, it also allows a very high SNR. Considering these features, the use of MPI for visualization and quantification of vascular pathologies like stenosis, aneurysms, anatomical variants and malformations plus functional assessment by perfusion measurements or imaging of vulnerable plaques are promising application scenarios. In current clinical practice, CT and MRI routinely cover those applications and many more like quantification of atherosclerotic plaques burden in CT. Besides the long established methods DSA and CCS, both methods are now recommended for many indications by international societies for cardiovascular medicine, e.g. peripheral artery disease [36], coronary artery disease [1, 37] or myocarditis [38]. However, both CT and MRI have drawbacks, like (high) doses of ionizing radiation, nephrotoxic contrast agents, impaired visualization of stenosis in stents and highly calcified vessels for CT and limited spatial and temporal resolution, limited patient access and restrictions for certain implants and medical devices for MRI. Current research addresses these issues: in CT, progress in reduction of ionizing radiation using iterative reconstruction [39, 40], visualization of vessel lumina along stents and calcifications [41, 42] and characterization of atherosclerotic plaques [43, 44] has been made. The development of free-breathing, ECG-triggered, navigator-gated, T2-prepared, 3-dimensional coronary MR angiography using steady state free precession (SSFP) sequence allows coronary imaging in MRI now, in principle without the need for contrast agents [45]. However, long examination times and limited spatial resolution are still an issue [45].

These few examples illustrate the highly competitive situation MPI faces in clinical imaging. However, if a very high temporal resolution can also be realized for human sized MPI systems, free breathing imaging sequences of the heart could be obtained in a matter of seconds without the need of ionizing radiation and perhaps even without ECG-triggering, which is not possible with MRI and CT. Given a sufficient spatial resolution, this could result in a fast assessment of the coronary vessels and measures like atrial and ventricular volumes and ejection fraction. Furthermore, MPI has the potential to visualize and quantify perfusion of organs, e.g. the brain and heart. Also, the visualization of vulnerable atherosclerotic plaques is feasible using SPIOs [27, 29]. Perfusion allows e.g. to assess the functional relevance of a vessel stenosis and the visualization of vulnerable atherosclerotic plaques is promising as those plaques bear a high risk of rupture and acute thrombotic vessel occlusion.

In contrast to MPI, CT and MRI provide anatomical information. As described above for vascular imaging and below for molecular imaging, this is not necessarily a drawback for MPI. On the other hand, the fusion of MRI or CT and MPI seems interesting and may prove beneficial, especially in perfusion-studies and cell tracking. MPI could provide quantitative and sensitive tracer images, if needed with a high frame rate, while MRI/CT would provide anatomical information with superb tissue contrast. As there are more similarities between MRI and MPI, i.e. both work with magnetic fields and without ionizing radiation and MRI provides the better soft tissue contrast than CT, the development of MPI/MRI-hybrid-systems or co-registration seems favorable and is already being pursued [46–48].

For cardiovascular MPI promising preclinical work has already been published [8, 49–51]. But to become an alternative in clinical imaging, MPI has to overcome challenges in hardware design and SPIO development: The FOV has to be enlarged to cover larger parts of the body like the thorax and the visualization of higher ranges of SPIO concentrations has to be realized. Furthermore, a spatial resolution of at least one millimeter in every direction should be obtained, although submillimeter spatial resolution is even more desirable for a detailed visualization of e.g. small vessel morphology. All that while preserving a sufficiently high temporal resolution. Also, patient safety has to be considered, especially regarding peripheral nerve stimulation (PNS) and patient heating (specific absorption rate, SAR) [30, 52, 53]. For image guidance of interventions, real-time imaging has to be combined with (quasi) real-time reconstruction [54]. First results towards real time MPI were already presented recently by Salamon et al. [55]. For MPI-guided interventions the safety of devices like guide wires and stents also has to be considered [56].

Until now, MPI hardware development proceeded to the realization of the first human sized MPI-system [5]. Initial results indicate that the achievable gradient strengths are comparable to the pre-clinical systems and thus similar spatial resolution can be expected. However, it is not yet established which temporal resolution can be achieved for a certain volume coverage. Imaging speed may be limited due to above mentioned effects of PNS and patient heating.

Besides hardware development, clinically available and approved SPIOs with improved MPI performance are crucial for use of MPI in humans. Resovist is still available in Japan, albeit the distribution in Europe has been discontinued and it has never been approved in the United States of America. Because of that and because Resovist is not an ideal MPI-SPIO, many working groups are developing MPI-dedicated SPIOs [21, 57], which should strongly improve the spatial resolution of MPI and allow to tailor the SPIOs pharmacokinetics for the specific application area. As a very promising example, Goodwill et al. already demonstrated cardiovascular MPI with a very high spatial resolution utilizing newly developed SPIOs, with the only drawback of a very long image acquisition time of 10 minutes [51]. A clinical approval process for MPI-SPIOs has not been started, yet. Thus and because currently no commercialization of a human sized MPI-system is foreseeable, human MPI will not be available in clinical routine soon. However, there are small animal models for e.g. atherosclerotic disease which may benefit from a reliable method to determine stenosis, organ perfusion and vulnerable atherosclerotic plaques, given an adequate temporal and spatial resolution. As various small animal MPI-systems are available and in use, preclinical approaches are already under investigation [8, 49, 51].

Molecular imaging is proposed to be another very interesting application for MPI. This is especially based on its high sensitivity in combination with a very good tissue penetration and noninvasiveness. As MPI detects electronic superparamagnetism in opposition to MRI, which detects nuclear paramagnetism, the magnetization detected by MPI is orders of magnitude higher than in MRI [9]. This explains the higher sensitivity of MPI for detection of SPIOs down to single digit nanograms of iron compared to micrograms in MRI. First promising results have been published for *in vitro* and *in vivo* cell labeling [58], tracking and targeting, e.g. *in vivo* visualization of the long term fate of neuroprogenitor cells in mice for three months [59]. If these results can be confirmed in further studies, MPI will be a valuable tool for preclinical imaging and will help to reduce the number of animals needed for longitudinal studies.

## Conclusion

This study shows that beside the fast and three-dimensional visualization of a vessel lumen, MPI allows direct quantification of stenoses down to SPIO-concentrations appropriate for clinical use. For clinical utilization, dedicated tracers are needed, spatial resolution and FOV have to be improved as well as the simultaneous visualization of highly different tracer concentrations while preserving high temporal resolution. The concept has to be proven in in vivo experiments. However, the results show that MPI already is a tool for quantitative imaging of SPIO-tracers, which can also be utilized in a preclinical context besides stenosis quantification, e.g. SPIO concentration of organs in small animal imaging or quantification of SPIOs in molecular imaging. Also, dynamic perfusion imaging is a further promising aspect of quantitative imaging.

## Supporting Information

S1 Table MPI Intensity measurements of the reference phantoms. Comparison of the cross sectional area of each reference phantom in relation to the area of the reference phantom with a diameter of 10 mm based on the known diameters on the one hand (2^nd^ and 3^rd^ column) and based on the MPI signal intensity measurements on the other hand (4^th^, 5^th^ and 6^th^ column). The SNR is given in S5 Table.
(DOCX)

S2 Table Known dimensions of the stenosis phantoms. Calculated absolute and relative cross sectional area of the stenosis and the degree of the stenosis of each stenosis phantom based on its known diameters.
(DOCX)

S3 Table MPI intensity measurements of the stenosis phantoms including the SNR. Measured MPI signal intensity values of the stenosis and the normal lumen of each stenosis phantom (2^nd^ and 3^rd^ column). Based on these values, the relative MPI signal intensity of the stenosis and the degree of the stenosis were calculated (4^th^ and 5^th^ column). The mean SNR is 570.52 (SD 76.36).
(DOCX)

S4 Table Resovist dilution series reconstructed with different regularization factors. Comparison of the calculated residual area and degree of the stenosis based on MPI signal intensity measurements of six different concentrations of Resovist using the 5 mm stenosis phantom using the regularization factors of λ = 1, λ = 10 and λ = 100. The calculated residual area and degree of the stenosis based on the known diameters of the stenosis phantoms are 75% and 25%, respectively. The SNR is shown in the right column. It can be seen that for the 1:3200 Resovist dilution with a λ = 1 the SNR is only 4.06, which leads to an underestimation of the degree of the stenosis. However, when the noise level is reduced due to the higher regularization factor of λ = 10, the quantification is as exact as with the higher concentrations of Resovist. With a regularization factor of λ = 100 the stenosis is overestimated due to the reduced spatial resolution.
(DOCX)

S5 Table Detailed signal to noise ratios of the reference phantoms. The signal to noise ratio is given for all reference phantoms. Additionally, the average I_MPI_ of the reference phantoms and the SD of the average noise per voxel from which the SNR is calculated are given. It can be seen that the noise level is relatively stable, but the average IMPI per voxel and consequently the SNR constantly decrease with diameters of the reference phantoms smaller than 6 mm.
(DOCX)

S6 Table Detailed signal to noise ratios of the dilution series. Corresponding to the S4 Table the signal to noise ratio is given for all dilutions and regularization factors of the stenosis phantom with a stenosis diameter of 5 mm. Additionally, the average I_MPI_ of the stenosis phantom and the SD of the average noise per voxel from which the SNR is calculated are given. With higher regularization the noise decreases. This leads to an increase of the SNR with higher regularization. The I_MPI_ decreases with higher Resovist dilutions whereas the noise is nearly constant within the respective regularization, resulting in decreasing SNR. As can be calculated based on the values of column 4, the mean SD of average noise per pixel for the different regularization factors are 1.89E-05 (±2.03E-06) for λ = 1; 6,45E-06 (±5,11E-07) for λ = 10 and 1,78E-06 (±1,55E-07) for λ = 100.
(DOCX)
