# Supplementary material for: Magnetic Particle Imaging (MPI): Experimental Quantification of Vascular Stenosis Using Stationary Stenosis Phantoms
Source: PLoS One. 2017 Jan 5;12(1):e0168902. doi: 10.1371/journal.pone.0168902 (PMC5215859; doi:10.1371/journal.pone.0168902)
Supplement: S1 Table — Comparison of the cross sectional area of each reference phantom in relation to the area of the reference phantom with a diameter of 10 mm based on the known diameters on the one hand (2nd and 3rd column) and based on the MPI signal intensity measurements on the other hand (4th, 5th and 6th column). The SNR is given in S5 Table. (DOCX) [file pone.0168902.s001.docx]

| Diameter (mm) | Area (mm^2^) | Area in relation to d = 10mm (%) | I_MPI_ | I_MPI_ in relation to d = 10 mm (%) | Degree of lumen loss in relation to d = 10 mm (%) |
| --- | --- | --- | --- | --- | --- |
| 10 | 78.54 | 100 | 0.88 | 100 | - |
| 9 | 63.62 | 81 | 0.71 | 81.28 | 18.72 |
| 8 | 50.27 | 64 | 0.58 | 65.95 | 34.05 |
| 7 | 38.48 | 49 | 0.44 | 49.85 | 50.15 |
| 6 | 28.27 | 36 | 0.33 | 37.69 | 62.31 |
| 5 | 19.63 | 25 | 0.23 | 26.42 | 73.58 |
| 4 | 12.57 | 16 | 0.15 | 17.25 | 82.75 |
| 3 | 7.07 | 9 | 0.09 | 9.84 | 90.16 |
| 2 | 3.14 | 4 | 0.04 | 4.59 | 95.41 |
| 1 | 0.79 | 1 | 0.01 | 1.22 | 98.78 |

**S1 Table. MPI Intensity measurements of the reference phantoms.** Comparison of the cross sectional area of each reference phantom in relation to the area of the reference phantom with a diameter of 10 mm based on the known diameters on the one hand (2^nd^ and 3^rd^ column) and based on the MPI signal intensity measurements on the other hand (4^th^, 5^th^ and 6^th^ column). The SNR is given in S5 Table.

I_MPI_ = MPI signal intensity (arbitrary units), d = diameter, mm = millimeter, mm^2^ = square millimeters, % = percent.
