# Supplementary material for: Magnetic Particle Imaging (MPI): Experimental Quantification of Vascular Stenosis Using Stationary Stenosis Phantoms
Source: PLoS One. 2017 Jan 5;12(1):e0168902. doi: 10.1371/journal.pone.0168902 (PMC5215859; doi:10.1371/journal.pone.0168902)
Supplement: S2 Table — Calculated absolute and relative cross sectional area of the stenosis and the degree of the stenosis of each stenosis phantom based on its known diameters. (DOCX) [file pone.0168902.s002.docx]

| Diameter of the stenosis (mm) | Area of the  stenosis (mm^2^) | Relative residual area of the stenosis^a^ (%) | Degree of stenosis (%) |
| --- | --- | --- | --- |
| 9 | 63.62 | 81 | 19 |
| 8 | 50.27 | 64 | 36 |
| 7 | 38.48 | 49 | 51 |
| 6 | 28.27 | 36 | 64 |
| 5 | 19.63 | 25 | 75 |
| 4 | 12.57 | 16 | 84 |
| 3 | 7.07 | 9 | 91 |
| 2 | 3.14 | 4 | 96 |
| 1 | 0.79 | 1 | 99 |

**S2 Table. Known dimensions of the stenosis phantoms.**

Calculated absolute and relative cross sectional area of the stenosis and the degree of the stenosis of each stenosis phantom based on its known diameters.

mm = millimeter, mm^2^ = square millimeters, % = percent; ^a^the relative residual area is described in relation to the area of the normal lumen (d = 10 mm) of each stenosis phantom.
