# Supplementary material for: Magnetic Particle Imaging (MPI): Experimental Quantification of Vascular Stenosis Using Stationary Stenosis Phantoms
Source: PLoS One. 2017 Jan 5;12(1):e0168902. doi: 10.1371/journal.pone.0168902 (PMC5215859; doi:10.1371/journal.pone.0168902)
Supplement: S3 Table — Measured MPI signal intensity values of the stenosis and the normal lumen of each stenosis phantom (2nd and 3rd column). Based on these values, the relative MPI signal intensity of the stenosis and the degree of the stenosis were calculated (4th and 5th column). The mean SNR is 570.52 (SD 76.36). (DOCX) [file pone.0168902.s003.docx]

| Diameter of the stenosis (mm) | I_MPI_ of the stenosis | I_MPI_ of the normal lumen d = 10 mm | Relative I_MPI_ of the stenosis^a^ (%) | Degree of stenosis^a^ (%) | Signal to noise ratio (SNR) |
| --- | --- | --- | --- | --- | --- |
| 9 | 0.721 | 0.907 | 79.531 | 20.469 | 429.37 |
| 8 | 0.567 | 0.912 | 62.164 | 37.836 | 593.77 |
| 7 | 0.415 | 0.915 | 45.308 | 54.692 | 624.33 |
| 6 | 0.308 | 0.934 | 33.013 | 66.987 | 579.40 |
| 5 | 0.210 | 0.936 | 22.408 | 77.592 | 608.51 |
| 4 | 0.128 | 0.945 | 13.499 | 86.501 | 560.07 |
| 3 | 0.070 | 0.949 | 7.389 | 92.611 | 450.96 |
| 2 | 0.023 | 0.961 | 2.423 | 97.577 | 609.86 |
| 1 | 0.014 | 0.977 | 1.413 | 98.587 | 678.44 |

**S3 Table. MPI intensity measurements of the stenosis phantoms including the SNR.**

Measured MPI signal intensity values of the stenosis and the normal lumen of each stenosis phantom (2^nd^ and 3^rd^ column). Based on these values, the relative MPI signal intensity of the stenosis and the degree of the stenosis were calculated (4^th^ and 5^th^ column). The mean SNR is 570.52 (SD 76.36).

I_MPI_ = MPI signal intensity (arbitrary units), d = diameter, mm = millimeter, mm^2^ = square millimeters, % = percent, SNR = signal to noise ratio; ^a^the relative MPI signal intensity and the degree of the stenosis are calculated in relation to the signal intensity of the normal lumen (d = 10 mm) of each stenosis phantom.
