# Supplementary material for: Magnetic Particle Imaging (MPI): Experimental Quantification of Vascular Stenosis Using Stationary Stenosis Phantoms
Source: PLoS One. 2017 Jan 5;12(1):e0168902. doi: 10.1371/journal.pone.0168902 (PMC5215859; doi:10.1371/journal.pone.0168902)
Supplement: S4 Table — Comparison of the calculated residual area and degree of the stenosis based on MPI signal intensity measurements of six different concentrations of Resovist using the 5 mm stenosis phantom using the regularization factors of λ = 1, λ = 10 and λ = 100. The calculated residual area and degree of the stenosis based on the known diameters of the stenosis phantoms are 75% and 25%, respectively. The SNR is shown in the right column. It can be seen that for the 1:3200 Resovist dilution with a λ = 1 the SNR is only 4.06, which leads to an underestimation of the degree of the stenosis. However, when the noise level is reduced due to the higher regularization factor of λ = 10, the quantification is as exact as with the higher concentrations of Resovist. With a regularization factor of λ = 100 the stenosis is overestimated due to the reduced spatial resolution. (DOCX) [file pone.0168902.s004.docx]

| **Resovist Dilution** | **Regularization factor λ** | **I_MPI_ of the stenosis** | **I_MPI_ of the normal lumen d = 10 mm** | **Relative I_MPI_ of the stenosis^a^ (%)** | **Degree of stenosis^a^ (%)** | **Signal to noise ratio (SNR)** |
| --- | --- | --- | --- | --- | --- | --- |
| **1:100** | 1 | 0.2097 | 0.936 | 22.41 | 77.59 | 454.96 |
|  | 10 | 0.1997 | 0.9098 | 21.89 | 78.11 | 1391.42 |
|  | 100 | 0.1460 | 0.7875 | 18.54 | 81.46 | 4115.47 |
| **1:200** | 1 | 0.0989 | 0.4723 | 20.94 | 79.06 | 268.28 |
|  | 10 | 0.1052 | 0.4766 | 22.07 | 77.93 | 821.08 |
|  | 100 | 0.0748 | 0.4178 | 17.89 | 82.01 | 2342.17 |
| **1:400** | 1 | 0.0496 | 0.2281 | 21.76 | 78.24 | 120.72 |
|  | 10 | 0.0523 | 0.233 | 22.45 | 77.55 | 344.85 |
|  | 100 | 0.0366 | 0.2102 | 17.39 | 82.61 | 1164.73 |
| **1:800** | 1 | 0.0261 | 0.1204 | 21.64 | 78.36 | 76.44 |
|  | 10 | 0.027 | 0.126 | 21.46 | 78.54 | 216.16 |
|  | 100 | 0.0177 | 0.1071 | 16.53 | 83.47 | 656.18 |
| **1:1600** | 1 | 0.0138 | 0.059 | 23.32 | 76.68 | 31.94 |
|  | 10 | 0.013 | 0.0624 | 20.89 | 79.11 | 96.91 |
|  | 100 | 0.0089 | 0.0535 | 16.58 | 83.42 | 272.47 |
| **1:3200** | 1 | 0.0096 | 0.0305 | 31.52 | 68.48 | 4.06 |
|  | 10 | 0.0068 | 0.0309 | 21.92 | 78.08 | 56.19 |
|  | 100 | 0.0047 | 0.0268 | 17.41 | 82.59 | 172.66 |

**S4 Table. Resovist dilution series reconstructed with different regularization factors.**

Comparison of the calculated residual area and degree of the stenosis based on MPI signal intensity measurements of six different concentrations of Resovist using the 5 mm stenosis phantom and the regularization factors of λ = 1, λ = 10 and λ = 100. The calculated residual area and degree of the stenosis based on the known diameters of the stenosis phantoms are 75 % and 25 %, respectively. The SNR is shown in the right column. It can be seen that for the 1:3200 Resovist dilution with a λ = 1 the SNR is only 4.06, which leads to an underestimation of the degree of the stenosis. However, when the noise level is reduced due to the higher regularization factor of λ = 10, the quantification is as exact as with the higher concentrations of Resovist. With a regularization factor of λ = 100 the stenosis is overestimated due to the reduced spatial resolution.

I_MPI_ = MPI signal intensity (arbitrary units), d = diameter, mm = millimeter, mm^2^ = square millimeters, % = percent; ^a^the relative MPI signal intensity and the degree of stenosis are described in relation to the signal intensity of the normal lumen (d = 10 mm) of each stenosis phantom.
