# Supplementary material for: Magnetic Particle Imaging (MPI): Experimental Quantification of Vascular Stenosis Using Stationary Stenosis Phantoms
Source: PLoS One. 2017 Jan 5;12(1):e0168902. doi: 10.1371/journal.pone.0168902 (PMC5215859; doi:10.1371/journal.pone.0168902)
Supplement: S5 Table — The signal to noise ratio is given for all reference phantoms. Additionally, the average IMPI of the reference phantoms and the SD of the average noise per voxel from which the SNR is calculated are given. It can be seen that the noise level is relatively stable, but the average IMPI per voxel and consequently the SNR constantly decrease with diameters of the reference phantoms smaller than 6 mm. (DOCX) [file pone.0168902.s005.docx]

| **Diameter (mm)** | **Average I_MPI_**  **per voxel** | **SD of average noise per voxel** | **Signal to noise ratio** |
| --- | --- | --- | --- |
| 10 | 9.80E-03 | 1.75E-05 | 559.29 |
| 9 | 9.92E-03 | 1.90E-05 | 522.82 |
| 8 | 9.74E-03 | 1.78E-05 | 547.50 |
| 7 | 9.61E-03 | 1.51E-05 | 638.02 |
| 6 | 9.55E-03 | 1.87E-05 | 511.15 |
| 5 | 8.50E-03 | 1.84E-05 | 462.30 |
| 4 | 6.76E-03 | 1.52E-05 | 443.88 |
| 3 | 4.66E-03 | 1.88E-05 | 247.93 |
| 2 | 2.46E-03 | 1.68E-05 | 146.89 |
| 1 | 7.91E-04 | 1.37E-05 | 57.81 |
| Mean | 7.18E-03 | 1.71E-05 | 413.76 |
| SD | 3.22E-03 | 1.75E-06 | 184.23 |

**S5 Table. Detailed signal to noise ratios of the reference phantoms.**

The signal to noise ratio is given for all reference phantoms. Additionally, the average I_MPI_ of the reference phantoms and the SD of the average noise per voxel from which the SNR is calculated are given. It can be seen that the noise level is relatively stable, but the average IMPI per voxel and consequently the SNR constantly decrease with diameters of the reference phantoms smaller than 6 mm, as the average I_MPI_ per voxel could not be measured sufficiently due to partial volume artifacts.

I_MPI_ = MPI signal intensity (arbitrary units), SD = standard deviation, SNR = signal to noise ratio.
