# Supplementary material for: Magnetic Particle Imaging (MPI): Experimental Quantification of Vascular Stenosis Using Stationary Stenosis Phantoms
Source: PLoS One. 2017 Jan 5;12(1):e0168902. doi: 10.1371/journal.pone.0168902 (PMC5215859; doi:10.1371/journal.pone.0168902)
Supplement: S6 Table — Corresponding to the S4 Table the signal to noise ratio is given for all dilutions and regularization factors of the stenosis phantom with a stenosis diameter of 5 mm. Additionally, the average IMPI of the stenosis phantom and the SD of the average noise per voxel from which the SNR is calculated are given. With higher regularization the noise decreases. This leads to an increase of the SNR with higher regularization. The IMPI decreases with higher Resovist dilutions whereas the noise is nearly constant within the respective regularization, resulting in decreasing SNR. As can be calculated based on the values of column 4, the mean SD of average noise per pixel for the different regularization factors are 1.89E-05 (±2.03E-06) for λ = 1; 6,45E-06 (±5,11E-07) for λ = 10 and 1,78E-06 (±1,55E-07) for λ = 100. (DOCX) [file pone.0168902.s006.docx]

| **Resovist Dilution** | **Regularization factor λ** | **Average I_MPI_**  **per voxel** | **SD of average noise per voxel** | **Signal to noise ratio (SNR)** |
| --- | --- | --- | --- | --- |
| **1:100** | 1 | 9.62E-03 | 2.11E-05 | 454.96 |
|  | 10 | 9.57E-03 | 6.88E-06 | 1391.42 |
|  | 100 | 7.91E-03 | 1.92E-06 | 4115.47 |
| **1:200** | 1 | 5.18E-03 | 1.93E-05 | 268.28 |
|  | 10 | 4.96E-03 | 6.04E-06 | 821.08 |
|  | 100 | 3.95E-03 | 1.69E-06 | 2342.17 |
| **1:400** | 1 | 2.52E-03 | 2.09E-05 | 120.72 |
|  | 10 | 2.52E-03 | 7.31E-06 | 344.85 |
|  | 100 | 2.07E-03 | 1.78E-06 | 1164.73 |
| **1:800** | 1 | 1.31E-03 | 1.71E-05 | 76.44 |
|  | 10 | 1.30E-03 | 5.99E-06 | 216.16 |
|  | 100 | 1.05E-03 | 1.61E-06 | 656.18 |
| **1:1600** | 1 | 6.31E-04 | 1.98E-05 | 31.94 |
|  | 10 | 6.33E-04 | 6.54E-06 | 96.91 |
|  | 100 | 5.55E-04 | 2.04E-06 | 272.47 |
| **1:3200** | 1 | 6.29E-05 | 1.55E-05 | 4.06 |
|  | 10 | 3.34E-04 | 5.95E-06 | 56.19 |
|  | 100 | 2.84E-04 | 1.64E-06 | 172.66 |

**S5 Table. Detailed signal to noise ratios of the dilution series.**

Corresponding to the S4­_Table the signal to noise ratio is given for all dilutions and regularization factors of the stenosis phantom with a stenosis diameter of 5 mm. Additionally, the average I_MPI_ of the stenosis phantom and the SD of the average noise per voxel from which the SNR is calculated are given. With higher regularization, the noise decreases. This leads to an increase of the SNR with higher regularization. The I_MPI_ decreases with higher Resovist dilutions whereas the noise is nearly constant within the respective regularization, resulting in a decrease of the SNR. As can be calculated based on the values of column 4, the mean SD of average noise per pixel for the different regularization factors are 1.89E-05 (±2.03E-06) for λ = 1; 6,45E-06 (±5,11E-07) for λ = 10 and 1,78E-06 (±1,55E-07) for λ = 100.

I_MPI_ = MPI signal intensity (arbitrary units), λ = regularization factor, SD = standard deviation, SNR = signal to noise ratio.
